# Early Postmortem Changes in NADH Fluorescence Lifetime Parameters in Rat Skeletal Muscle: Potential for Postmortem Interval Estimation

**DOI:** 10.3390/ijms27188094

**Published:** 2026-09-11

**Authors:** Anastasiya Babkina, Ivan Ryzhkov, Mikhail Yadgarov, Elena Potapova, Viktor Dremin, Ksenia Kandurova, Evgeniya Seryogina, Valery Shupletsov, Arkady Golubev, Dmitry Sundukov, Artem Kuzovlev

**Affiliations:** 1 Federal Research and Clinical Center of Intensive Care Medicine and Rehabilitology, 107031 Moscow, Russia; riamed21@gmail.com (I.R.); myadgarov@fnkcrr.ru (M.Y.); arkadygolubev@mail.ru (A.G.); artem_kuzovlev@fnkcrr.ru (A.K.); 2 Department of Forensic Medicine, Institute of Medicine, Peoples’ Friendship University of Russia (RUDN University), 117198 Moscow, Russia; sundukov.1958@mail.ru; 3 Research & Development Center of Biomedical Photonics, Orel State University, 302026 Orel, Russia; potapova_ev_ogu@mail.ru (E.P.); dremin_viktor@mail.ru (V.D.); k.kandurova@oreluniver.ru (K.K.); e.seryogina@oreluniver.ru (E.S.); valery.shupletsov@bmecenter.ru (V.S.)

**Keywords:** NADH autofluorescence, time-resolved fluorescence spectroscopy, postmortem interval estimation, skeletal muscle, postmortem metabolism, forensic medicine, optical spectroscopy, biophotonics

## Abstract

Investigation of the molecular mechanisms of postmortem processes to identify postmortem interval (PMI) markers is highly relevant in forensic medicine, given the insufficient accuracy and substantial limitations of current routine PMI estimation methods. This exploratory study aimed to describe changes in NADH fluorescence decay parameters in rat skeletal muscle during the early postmortem period and to assess their potential for PMI estimation. Using time-resolved fluorescence spectroscopy, the short- and long-lifetime components (τ_1_ and τ_2_), the amplitude-weighted mean fluorescence lifetime (τ_m_), the relative contributions of the short- and long-lifetime components (α_1_ and α_2_, respectively) and fluorescence intensity were measured in 10 Wistar rats before death, immediately after death, every 30 min during the first 6 h, and at 24 h postmortem. Immediately after death, τ_1_ decreased compared with the antemortem value (*p* = 0.02). τ_1_, τ_m_, α_1_, and α_2_ were dependent on the postmortem interval. For PMI prediction within the first 6 h after death, a partial least squares regression model combining α_1_, τ_m_, and the difference between rectal and room temperatures was constructed. The model demonstrated promising predictive performance, with a root mean square error (RMSE) of 0.89 h (95% CI: 0.63–0.99), a mean absolute error (MAE) of 0.68 h (95% CI: 0.49–0.82) and an R^2^ of 0.762 (95% CI: 0.689–0.886); the prediction error did not exceed 1 h in 73.2% of cases. These data are preliminary and require confirmation in studies with larger samples under different ambient temperature conditions and subsequent validation on human cadaveric material.

## 1. Introduction

The development of methods for estimating the postmortem interval (PMI) is one of the key areas of research in forensic medicine. The routine methods applied in forensic practice, which are based on the assessment of postmortem cooling (algor mortis), muscular rigidity (rigor mortis) and postmortem lividity (livor mortis), have substantial limitations. Despite the wide range of approaches available for PMI estimation in the early postmortem period, the assessment of postmortem changes and supravital reactions, particularly within the first hours after death, is associated with certain difficulties: interindividual variability in the onset and fixation of postmortem lividity, the development of rigor mortis, and the rate of postmortem cooling, as well as the initial temperature plateau observed in a number of cases [1,2,3]. Changes associated with tissue supravitality are used as additional criteria for PMI estimation during the early postmortem period [4]. Some supravital reactions, including the mechanical and electrical excitability of skeletal muscle and the response of the iris to pharmacological agents, are of practical value in forensic medicine and are applied in combination with other conventional methods of PMI estimation. However, the methods used to assess supravital reactions are associated with considerable uncertainty for several reasons: the magnitude and duration of skeletal muscle responses depend on the muscle examined, the mode and intensity of stimulation, the examiner’s experience, and the individual characteristics of the deceased [5,6]. The pharmacological response of the iris is observed in only a proportion of cadavers, does not show a consistent dependence on the PMI, and may be accompanied by paradoxical changes in pupil diameter [7,8,9].

Because of the limitations of the methods outlined above, current research increasingly focuses on identifying molecular markers of postmortem processes. Recent studies have highlighted the potential of time-dependent molecular changes in skeletal muscle as markers for PMI estimation, including changes in protein degradation, energy metabolism, and transcriptional profiles [10,11]. Structural, metabolic, and transcriptional changes were dependent on ambient temperature, whereas postmortem degradation patterns of certain proteins were reported to be little influenced by environmental conditions but varied depending on muscle type, the presence of lesions, and intra- and interindividual factors. These findings highlight the potential value of further investigating PMI-dependent molecular changes in skeletal muscle. Accordingly, the development of methods for estimating PMI in the early postmortem period requires a fundamental understanding of tissue metabolism and the mechanisms underlying tissue survival under hypoxic and anoxic conditions.

The reduced form of nicotinamide adenine dinucleotide (NADH), which accumulates under hypoxic conditions, exhibits autofluorescence, and the parameters of its autofluorescence can therefore be used as indicators of tissue metabolism [12,13].

Despite the widespread use of NADH autofluorescence in biomedical research, the possibility of using this coenzyme for PMI estimation has been addressed in only a few studies. Donaldson et al. were the first to demonstrate a time-dependent increase in NADH concentration in the blood of deceased individuals, as well as in blood stored in vacuum tubes, over 96 h [14]. Alaa El-Din et al. likewise reported a PMI-dependent increase in blood NADH concentration, which the authors attributed to degenerative changes in neutrophils [15].

Gruszczyńska et al. proposed a novel non-invasive spectroscopic approach to PMI assessment based on the previously undescribed excitation energy transfer between free tryptophan and free NADH in fingerprint sweat–fatty substances, with NADH fluorescence changes considered as potential postmortem markers [16].

In our previous experimental studies, fluorescence spectroscopy revealed a characteristic time course of NADH fluorescence intensity changes in rat skeletal muscle during the early postmortem period, consisting of an increase in fluorescence intensity during the first 3 h of anoxia followed by a gradual decline towards the end of the first 24 h [17].

In contrast to fluorescence intensity measurements, fluorescence lifetime analysis does not rely on the absolute recorded fluorescence intensity and is therefore less affected by variations in light absorption and scattering, which is particularly important for fluorescence measurements in biological tissues. In addition, fluorescence decay analysis provides information on the relative contributions of free and protein-bound NADH and on changes in its molecular environment [18]. This makes fluorescence decay parameters suitable for the assessment of the metabolic state of tissues [19,20,21,22,23] and supports the potential of time-resolved methods for investigating the molecular mechanisms of postmortem changes in tissues [24]. In a biexponential model, the short-lifetime component τ_1_ is predominantly associated with free NADH, whereas the long-lifetime component τ_2_ is predominantly associated with protein-bound NADH. The parameters α_1_ and α_2_ reflect the relative contributions of the corresponding components to the recorded fluorescence decay, while the amplitude-weighted mean fluorescence lifetime τ_m_ is a composite parameter that depends simultaneously on the values of τ_1_ and τ_2_ and on their relative contributions [25].

The development of time-resolved fluorescence spectroscopy (FS) techniques has substantially expanded the possibilities for studying metabolic changes in hypoxia, ischemia and mitochondrial dysfunction. In the context of early PMI estimation, this approach may complement conventional methods based on postmortem changes and supravital reactions by enabling quantitative assessment of postmortem metabolic changes in tissues. Despite the widespread use of time-resolved FS in basic and applied biomedical research, to the best of our knowledge, NADH fluorescence decay parameters have not been evaluated as potential markers for PMI estimation.

The aim of this exploratory study was to describe changes in NADH fluorescence decay parameters in rat skeletal muscle during the early postmortem period and to assess their potential for PMI estimation.

## 2. Results

### 2.1. NADH Fluorescence Parameters in Rat Skeletal Muscle Immediately After Death Compared with Antemortem Values

Compared with the antemortem values, a significant decrease in the short-lifetime component of NADH fluorescence (τ_1_) was observed immediately after death (*p* = 0.020). The remaining fluorescence parameters, including the long-lifetime component of NADH fluorescence (τ_2_), the amplitude-weighted mean fluorescence lifetime (τ_m_), the relative contributions of the short- and long-lifetime components (α_1_ and α_2_, %), and the fluorescence intensity, did not differ significantly (Table 1).

### 2.2. Postmortem Changes in NADH Fluorescence Parameters and Temperature

The individual values of the fluorescence parameters, the rectal temperature, and the surface temperature of the limb on which the measurements were performed are presented for each time point in Table S1 of the Supplementary Materials. Descriptive statistics for the parameters studied are presented in Table A1 of Appendix A.

The measurements were performed at a mean laboratory air temperature of 24.3 °C (range, 21.7–26.3 °C) and a relative humidity of 32–35%. The median rectal temperature decreased from 35.3 °C to 27.1 °C over the postmortem period from 0 to 6 h, and the median hind-limb temperature decreased from 31.1 °C to 24.9 °C over the same period.

**Figure 1:**
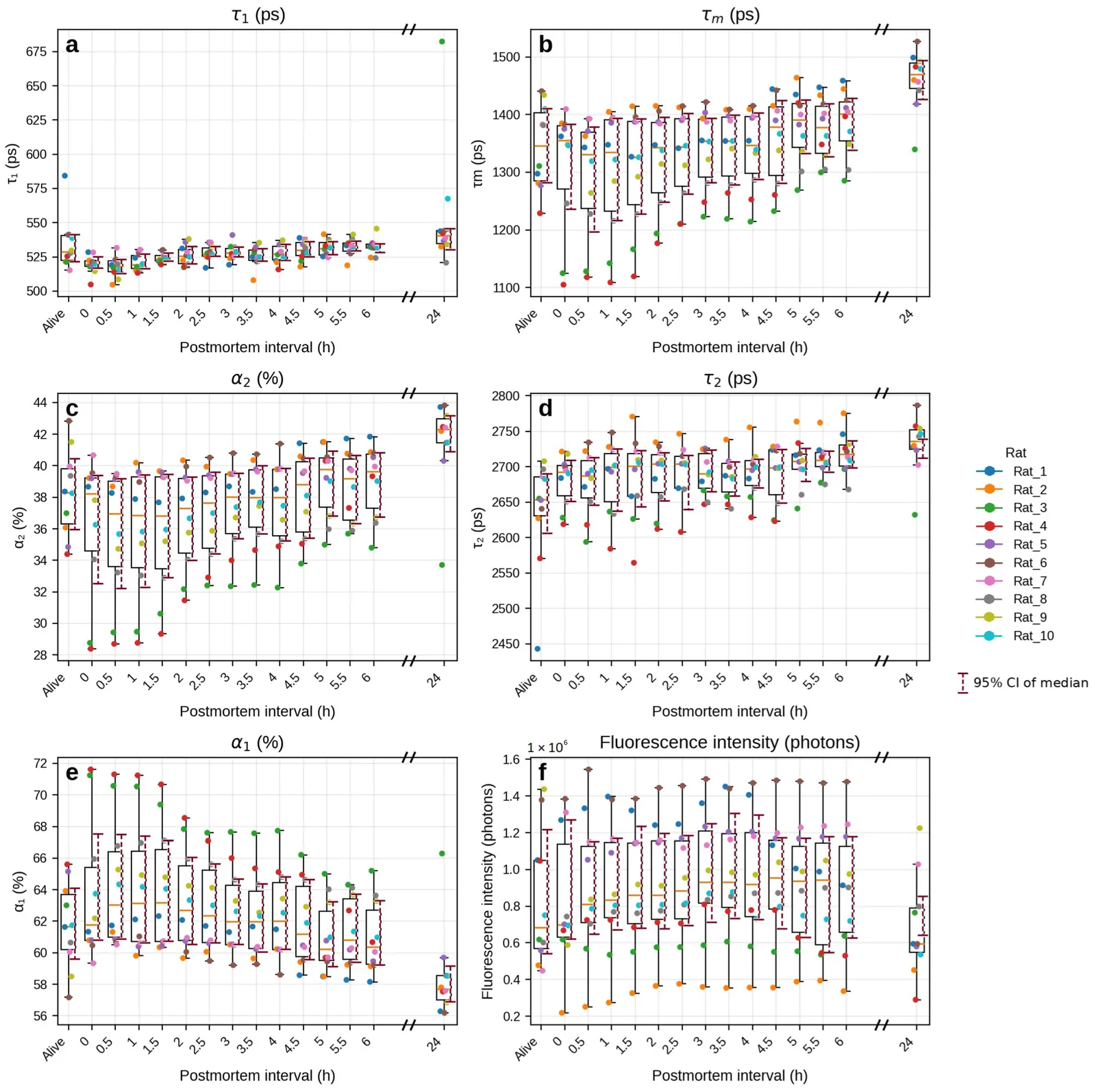
The distributions of NADH fluorescence parameters across the postmortem time points: (**a**) τ_1_ (ps); (**b**) τ_m_ (ps); (**c**) α_2_ (%); (**d**) τ_2_ (ps); (**e**) α_1_ (%); (**f**) fluorescence intensity (photons). Dark-red dashed brackets indicate the 95% percentile bootstrap CI for the median.

The distributions of the NADH fluorescence parameters across the postmortem time points are shown in Figure 1. Descriptive analysis of the median values indicated an increase in τ_1_ starting at 1.5 h (523.1 ps), reaching a maximum at 24 h (536.3 ps) (Figure 1a). The median value of τ_2_ increased from 3.5 h (2686.7 ps) to 24 h (2727.7 ps) (Figure 1d). The median value of τ_m_ remained below the median at the 0 h time point throughout the first 4 h of the postmortem period; from 4 h to 24 h, the median values increased from 1346.8 ps to 1472.4 ps (Figure 1b). Changes in the median relative contributions of the short- and long-lifetime components became more pronounced from 2 h postmortem onward and were characterized by an increase in α_2_ to 42.23% and a corresponding decrease in α_1_ to 57.77% at 24 h (Figure 1c,e). The median fluorescence intensity increased during the first hours after death, reaching a maximum at 4.5 h, and subsequently declined by 24 h (Figure 1f). The fluorescence parameters obtained from the right and left gastrocnemius muscles at 24 h did not differ significantly, except for τ_1_, for which a nominally significant difference was observed (unadjusted *p* = 0.049) (Table A2, Appendix A).

**Figure 2:**
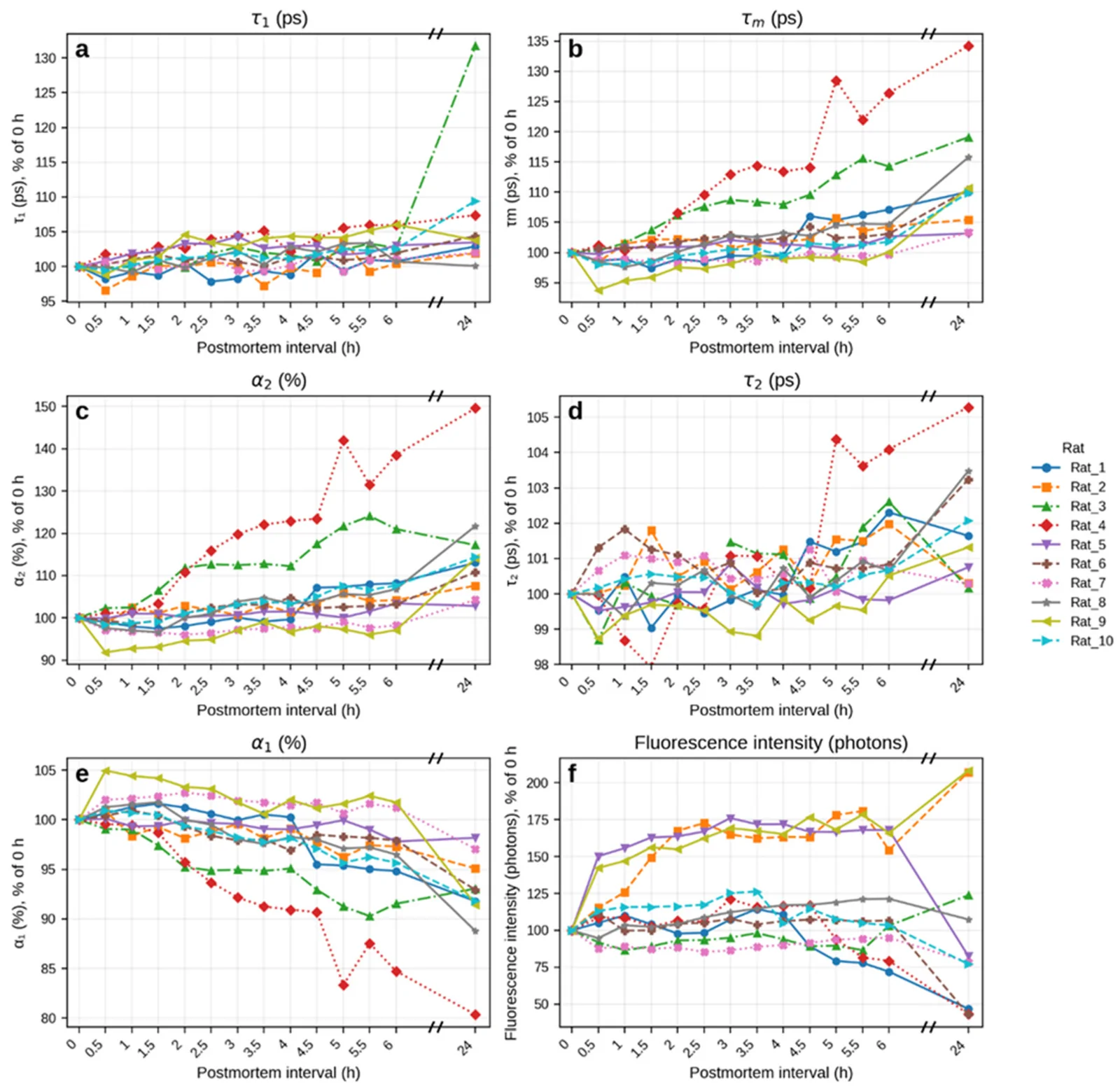
Individual relative changes in NADH fluorescence parameters during the postmortem period: (**a**) τ_1_ (ps); (**b**) τ_m_ (ps); (**c**) α_2_ (%); (**d**) τ_2_ (ps); (**e**) α_1_ (%); (**f**) fluorescence intensity (photons).

The individual relative changes in NADH fluorescence parameters during the postmortem period are presented in Figure 2. The values obtained immediately after death (0 h) were taken as 100% for each animal. The individual trajectories demonstrated variability in the magnitude and time course of changes in the fluorescence decay parameters during the early postmortem period. However, in most animals, an increase in τ_1_, τ_2_, and τ_m_ was observed by 6 h postmortem, accompanied by an increase in the relative contribution of the long-lifetime component (α_2_) and a decrease in the contribution of the short-lifetime component (α_1_) (Figure 2a–e). At 24 h after death, the values of τ_m_ and α_2_ (Figure 2b,c) exceeded the baseline values in all animals, whereas the values of α_1_ (Figure 2e) were below baseline in all animals. Fluorescence intensity showed the greatest interindividual variability, with different directions and magnitudes of change observed in individual animals (Figure 2f).

### 2.3. Relationship Between NADH Fluorescence Parameters and Postmortem Temperature Changes

τ_1_ showed significant negative partial correlations with rectal temperature (r = −0.260, *p* = 0.003), hind-limb surface temperature (r = −0.407, *p* < 0.001), and room temperature (r = −0.392, *p* < 0.001). Fluorescence intensity also correlated negatively with rectal, hind-limb surface, and room temperature. τ_m_, α_2_, τ_2_, and α_1_ showed no significant partial correlations with rectal temperature, hind-limb surface temperature, or room temperature after controlling for postmortem interval (Table 2).

### 2.4. Predictive Performance of NADH Fluorescence Parameters in Postmortem Interval Estimation

In the 0–24 h analysis, the best-performing individual predictor of PMI was τ_1_ (R^2^ = 0.267, 95% CI: 0.111–0.478; MAE = 2.80 h, 95% CI: 2.20–3.18). τ_m_ showed lower predictive value (R^2^ = 0.207, 95% CI: 0.132–0.376; MAE = 3.38 h, 95% CI: 3.08–3.45). Similar performance was observed for α_1_ (%) and α_2_ (%), whereas fluorescence intensity demonstrated minimal predictive ability (R^2^ = 0.012, 95% CI: 0.000–0.067).

The partial least squares (PLS) model based on α_1_ (%) and τ_m_ demonstrated limited performance over the full 0–24 h interval (R^2^ = 0.209, 95% CI: 0.149–0.394; MAE = 3.38 h, 95% CI: 3.03–3.46), with only 26 of 140 predictions (18.6%) falling within 1 h of the observed PMI (Table 3).

**Figure 3:**
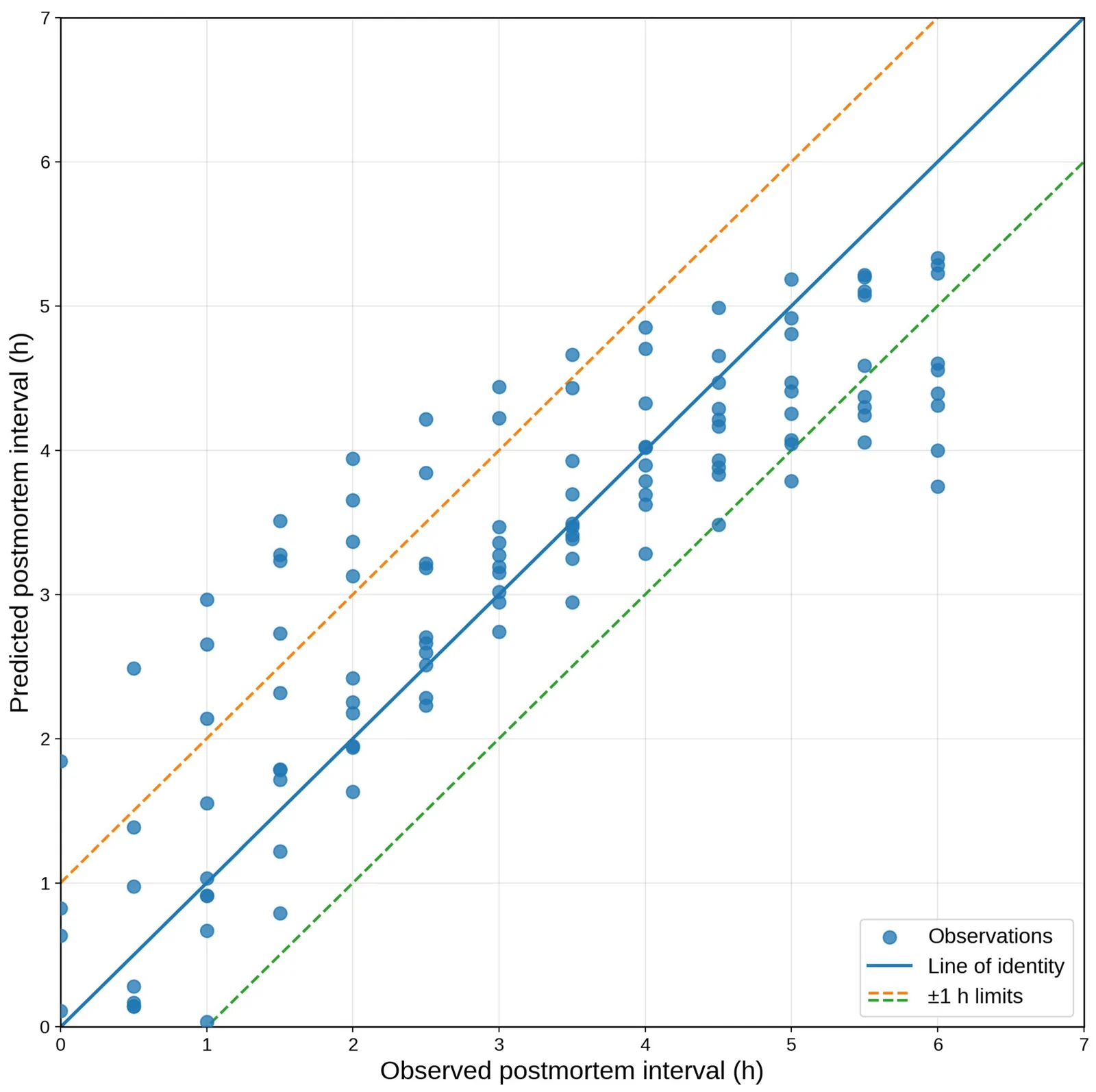
Observed versus predicted postmortem interval using the combined PLS model.

When the analysis was restricted to the 0–6 h postmortem interval, τ_1_ remained the best-performing individual predictor, with R^2^ = 0.319 (95% CI: 0.173–0.509), MAE = 1.28 h (95% CI: 1.05–1.46), and RMSE = 1.54 h (95% CI: 1.31–1.70). The PLS optical model showed similar performance to that of τ_m_ alone. In contrast, the combined PLS model incorporating α_1_, τ_m_, and the difference between rectal and room temperatures (ΔT) showed substantially better predictive performance (*n* = 123), achieving R^2^ = 0.762 (95% CI: 0.689–0.886), MAE = 0.68 h (95% CI: 0.49–0.82), and RMSE = 0.89 h (95% CI: 0.63–0.99). Overall, 90 of 123 predictions (73.2%) were within 1 h of the observed PMI (Table 4). The agreement between observed and predicted PMI for the combined model is shown in Figure 3.

## 3. Discussion

The present study is the first to describe the pattern of changes in NADH fluorescence decay parameters in skeletal muscle during the early postmortem period. These findings extend our current understanding of the biochemical changes that occur in skeletal muscle under conditions of anoxia and identify candidate markers for postmortem interval estimation. The parameters τ_1_ and τ_m_, as well as the relative contributions of the short- and long-lifetime components of biexponential fluorescence decay, α_1_ and α_2_, showed time-dependent changes during the early postmortem period. τ_1_ demonstrated the strongest predictive performance among the individual fluorescence parameters. The preliminary results of PLS modeling indicate the potential predictive value of a model combining fluorescence decay parameters with the difference between rectal and ambient temperatures for PMI estimation within the first 6 h after death.

According to the results of our study, the fluorescence lifetime of the short-lifetime component τ_1_, which is attributed predominantly to free NADH, decreased significantly immediately after death and showed the best predictive performance for PMI estimation. The changes identified may indicate that the short-lifetime NADH pool and its molecular microenvironment are particularly sensitive to the cessation of oxygen delivery. One of the principal factors triggering changes in energy metabolism and the cascade of postmortem changes is the progressive decrease in tissue oxygen content. Oxygen deficiency limits NADH oxidation, leading to the accumulation of reduced pyridine nucleotides. In our previous studies, an increase in NADH fluorescence intensity in rat skeletal muscle was observed from the fifth minute after death and continued up to 3 h of the postmortem period [26]. Richmond et al. showed that the increase in NADH fluorescence in rat skeletal muscle in vivo begins when the interstitial oxygen tension falls to a critical level at which point aerobic metabolism becomes limited [27]. Priest et al. demonstrated an increase in the ratio of free to protein-bound NADH during in vivo muscle contraction and interpreted these changes as an increase in the level of free NADH and an enhancement of glycolysis [28].

In our study, a decrease in the relative contribution of the short-lifetime component of the biexponential decay, α_1_, accompanied by an increase in the contribution of the long-lifetime component, α_2_, was observed from 2 h postmortem onward. These changes may be associated with ATP depletion and the onset of irreversible changes leading to the development of rigor mortis and loss of tissue viability. During the early postmortem period, ATP resynthesis is maintained through the creatine kinase reaction and anaerobic glycolysis, preserving skeletal muscle viability for a certain period [29,30]. The duration of the period of tissue viability, during which the possibility of functional recovery is retained, is considerably shorter than the duration of the supravital period [31]. In human skeletal muscle, the critical duration of complete ischemia depends on muscle temperature and amounts to approximately 2.25 h at a temperature of 34 °C. Irreversible injury begins after approximately 3 h and becomes almost complete by 6 h [32]. In contrast, the supravital electrical excitability of skeletal muscle may persist for up to 20 h postmortem [31]. According to Shintaku et al., the maximum duration of postmortem electrical excitability of the rat gastrocnemius muscle ranged from 60 to 110 min depending on temperature [33]. Calder et al. reported that intensive postmortem glycogenolysis occurs during the first two hours of the postmortem period, accompanied by the breakdown of approximately 82% of the initial glycogen, an increase in lactate, and a decrease in pH [34].

According to the results obtained, by 24 h postmortem, the mean NADH fluorescence lifetime and the relative contribution of the long-lifetime component had increased. Changes in NADH fluorescence lifetime may reflect not only alterations in the NAD(H) redox state but also changes in the total NAD(H) pool size, which can vary independently of the redox state. Song et al. demonstrated an inverse relationship between NAD(H) pool size and mean NADH fluorescence lifetime, with a decrease in pool size being accompanied by an increase in mean lifetime [18]. Thus, the increase in τ_m_ observed in our study by 24 h postmortem may partly reflect a decrease in the NAD(H) pool size, although this interpretation requires direct experimental confirmation.

Several postmortem biochemical processes may also contribute to the observed increase in mean NADH fluorescence lifetime and the relative contribution of the long-lifetime component. Taking into account the progressive postmortem decrease in pH in skeletal muscle demonstrated in several experimental studies [35,36], its potential influence on NADH fluorescence decay parameters should be considered. Studies in cell cultures demonstrate an influence of pH on the fluorescence decay parameters. Schmitz et al. found an increase in the mean lifetime of the coenzymes upon acidification of the medium. However, the changes in the fluorescence parameters depended on the cell type. In some cell lines, the variation in the mean lifetime with extracellular pH was determined predominantly by changes in the relative contribution of the short-lifetime component, whereas in others, it was determined by a change in τ_1_ itself [37]. An increase in the mean NADH lifetime upon acidification of the intracellular medium was demonstrated in the study of Ogikubo et al. [38]. Cannon et al. reported that, within the pH range of 5–10, the direct effect of acidity on the decay parameters is relatively small and becomes more pronounced at pH values below 5 [39]. Anaerobic glycolysis that continues after death is accompanied by the accumulation of lactate and H^+^, which may affect both the fluorescence properties directly and the activity of cellular enzymes and the pattern of NADH binding to proteins, in particular to lactate dehydrogenase. It has been shown that lactate dehydrogenase activity persisting in skeletal muscle during the early postmortem period may sustain the formation of NADH; however, the influence of this process on the proportion of protein-bound NADH requires direct experimental confirmation [40,41].

The increase in the contribution of the long-lifetime forms of NADH may be associated with the preservation of the protein-bound NADH pool and accumulation of NADH on mitochondrial enzymes, as a consequence of the cessation of oxygen supply and inhibition of the respiratory chain [42,43,44]. According to time-resolved spectroscopic imaging data, mitochondrial protein-bound NADH accounts for approximately 70% of the total cellular NADH fluorescence signal [25]. Mitochondria may retain or restore key functions for hours after death, although these functions decrease with increasing PMI [42]. Cytochrome c oxidase (complex IV of the respiratory chain) activity also undergoes time-dependent postmortem changes and is regarded as a marker for PMI estimation [45]. Lv et al. established a strong positive linear relationship between mitochondrial membrane potential (ΔΨm) and PMI in the brain, myocardium, and skeletal muscle during the first 15–18 h after death [46]. In experimental models, interventions that alter respiratory chain function and the bioenergetic state of mitochondria are accompanied by systematic changes in NADH fluorescence parameters [47]. In particular, in a cellular model, exposure to rotenone led simultaneously to a decrease in ΔΨm, a shortening of the mean NADH fluorescence lifetime, and an increase in the relative contribution of the short-lifetime component [48]. The postmortem increase in ΔΨm reported by Lv et al. may therefore be consistent with the increase in mean NADH fluorescence lifetime observed in our study. However, the contribution of postmortem changes in mitochondrial function, including ΔΨm, to the observed changes in NADH fluorescence parameters remains hypothetical, since this relationship has not been investigated directly in postmortem skeletal muscle.

The changes in NADH fluorescence parameters observed in our study may also be influenced by postmortem alterations in Ca^2+^ homeostasis. Postmortem ATP depletion impairs Ca^2+^ removal from the sarcoplasm, leading to an increase in free Ca^2+^ concentration [49]. Ca^2+^ entry into mitochondria activates pyruvate dehydrogenase, NAD^+^-dependent isocitrate dehydrogenase, and the α-ketoglutarate dehydrogenase complex, promoting the formation of NADH. In intact rat cardiac trabeculae, an increase in mitochondrial Ca^2+^ was accompanied by an increase in NADH fluorescence [50,51,52].

In the present study, statistically significant associations with rectal, hind-limb surface, and ambient temperatures were identified for fluorescence intensity and τ_1_, but not for the other parameters examined. Temperature exerts both a direct effect on the optical properties of NADH and an indirect effect through changes in the rate of biochemical reactions; in addition, the dynamics of cooling are closely related to the duration of the postmortem period. According to Cannon et al., an increase in temperature (25–45 °C) was accompanied by a decrease in fluorescence intensity and in the amplitude-weighted mean lifetime τ_m_. The change in τ_1_ was comparatively small and variable, whereas τ_2_ and τ_m_ demonstrated a more pronounced temperature-related shift [39]. These findings only partly correspond to the results of the present study. Martinez et al. showed that an increase in temperature raises the proportion of free NADH not only in the LDH–NADH system but also in MCF-7 cells and primary neurons, which the authors attributed to a weakening of coenzyme binding to enzymes [53]. Extrapolation of these results to postmortem skeletal muscle requires caution, since the studies of Cannon et al. were performed in solutions, and the cellular models of Martinez et al. do not reproduce the complex processes occurring in cooling tissue after death.

Thus, the earliest changes in the fluorescence parameters during the postmortem period may be related to the persisting activity of anaerobic glycolysis. The subsequent increase in the mean fluorescence lifetime τ_m_ and in the relative contribution of the long-lifetime component α_2_ may initially reflect the preservation of the protein-bound NADH pool and its possible accumulation on mitochondrial enzymes as a consequence of inhibition of the respiratory chain. At later stages, these changes may reflect the combined influence of a pronounced decrease in pH, an increase in intracellular Ca^2+^ concentration, ATP depletion, and the development of irreversible structural and metabolic disturbances, including the formation of rigor mortis. At the same time, the interpretation of the results obtained is limited by the absence of comparable studies of skeletal muscle during the postmortem period.

The main contribution of the present study is the identification of NADH fluorescence decay parameters that show time-dependent changes during the early postmortem period. In particular, τ_1_ demonstrated the strongest predictive performance among the individual fluorescence parameters, whereas the changes in τ_m_ and in the relative contributions α_1_ and α_2_ indicate that different components of NADH fluorescence decay may provide complementary information on postmortem metabolic changes. In addition, the results of PLS modeling suggest that combining fluorescence decay parameters with temperature variables may be useful for the development of multivariable approaches to PMI estimation. Thus, the present findings provide a basis for selecting the most informative fluorescence parameters for further experimental and validation studies.

The present study has a number of limitations. A formal a priori power calculation was not performed because of the exploratory nature of the work; the results should therefore be regarded as preliminary. The small sample size was also determined by adherence to the Reduction principle of the 3Rs. Mixed-effects or repeated-measures modeling of the partly nonlinear trajectories was not performed because the study included only 10 animals, making estimation of random effects together with nonlinear time terms potentially unstable. Similarly, leave-one-animal-out or other grouped cross-validation was not used because each training set would contain only nine animals, resulting in highly variable performance estimates; these approaches should be evaluated in larger validation studies.

The probe was inserted within a single anatomical region of the muscle; however, its exact position relative to the individual components of the muscle tissue was not verified. The skin was not incised in order to prevent drying of the tissue and diffusion of oxygen from the external environment; however, this limited visual control of the probe position.

The study was conducted at room temperature and in only one skeletal muscle; therefore, the results cannot be directly extrapolated to other temperature conditions or to muscles with different metabolic characteristics. The possible effects of sex, body weight, and muscle composition on tissue metabolism and NADH fluorescence parameters were not evaluated in this study and should be addressed in further research.

The fixed body position did not allow for the assessment of the development and resolution of rigor mortis, and the absence of measurements between 6 and 24 h limited characterization of the time course of the parameters during this period.

## 4. Materials and Methods

### 4.1. Study Design

This was a prospective, exploratory, single-group experimental animal study with repeated within-animal measurements. Antemortem values obtained in animals under anesthesia before euthanasia were used as the reference for assessing the changes occurring immediately after death, whereas the values measured at 0 h served as the baseline control for all subsequent postmortem measurements. The experimental unit was an individual animal (rat). All animals underwent the same experimental protocol, and measurements were repeated in the same animal at predefined time points before death, immediately after death, every 30 min throughout the first 6 h, and at 24 h postmortem. Because the study was exploratory, no a priori sample size calculation was performed. Since only one experimental group was studied and all animals received identical procedures, randomization and blinding were not applicable. The inclusion criterion was clinically healthy status of the animal, established by veterinary examination before the experiment; no exclusion criteria were defined a priori. All 10 animals completed the protocol, and no animals or data points were excluded from the analysis.

### 4.2. Animal Model

The study was performed on male Wistar rats (*n* = 10) weighing 350–450 g. The age of the animals at the time of the experiment was 4 months. To limit biological variability in this exploratory study, the experimental cohort was restricted to male rats of the same age and a defined body-weight range. The animals were conventional (not specific pathogen-free), had not undergone any previous experimental procedures, were not genetically modified, and were clinically healthy on veterinary examination prior to the experiment. The animals were acclimatized to the housing conditions of the animal facility for 5 days before the start of the experiment. The animals were housed in standard cages, two per cage, at a temperature of 23 ± 1 °C and a humidity of 50 ± 10%, under a 12:12 h light–dark cycle. The animals had access to water and standard rodent chow ad libitum. All animal experiments were approved by the Local Ethics Committee of the Federal Research and Clinical Center of Intensive Care Medicine and Rehabilitology of the Ministry of Education and Science of the Russian Federation (protocol 3/26/5, approval date: 29 April 2026) and were conducted in accordance with Directive 2010/63/EU of the European Parliament and of the Council on the protection of animals used for scientific purposes and with the ARRIVE guidelines. Every effort was made to minimize animal suffering and to reduce the number of animals used.

### 4.3. Anesthesia and Euthanasia

All antemortem experimental procedures were performed under inhalation anesthesia with isoflurane at a working concentration of 1.3 vol%, delivered first in a transparent induction chamber and then through a face mask of a dedicated anesthesia machine (RWD R540 Mice & Rat Animal Anesthesia Machine; RWD Life Science Co., Ltd.,Shenzhen, China). The animal was secured in the prone position. Before the measurements, the fur in the optical probe insertion area was carefully shaved. After the antemortem NADH fluorescence decay spectra had been recorded, the rat, while still under general anesthesia, was euthanized by intracardiac injection of 2% lidocaine solution. Anesthesia was maintained continuously from induction until euthanasia so that all invasive procedures, including probe insertion, were performed in the unconscious animal; the depth of anesthesia was monitored based on respiratory rate and absence of the pedal withdrawal reflex.

### 4.4. Thermometry

Throughout the entire observation period, a rectal temperature probe of a surgical warming and monitoring system (Rodent Surgical Monitor S; Indus Instruments, Webster, TX, USA) was inserted into the rectum of the rat. Rectal temperature and the surface temperature of the hind limb were recorded at each time point until equilibration with the ambient temperature. The temperature of the hind limb was measured with a Sensitec NB-401 infrared thermometer (Shenzhen Everbest Machinery Industry Co., Ltd., Shenzhen, China). Ambient temperature and humidity were recorded at each time point with a portable weather station (Mijia Temperature and Humidity Sensor 2; MiaoMiaoCe Technology (Beijing) Co., Ltd.,Beijing, China). No active warming was applied at any stage of the experiment: the animals cooled passively at room temperature, since the rate of postmortem cooling was one of the variables under study.

### 4.5. Measurement of NADH Fluorescence Decay Spectra

**Figure 4:**
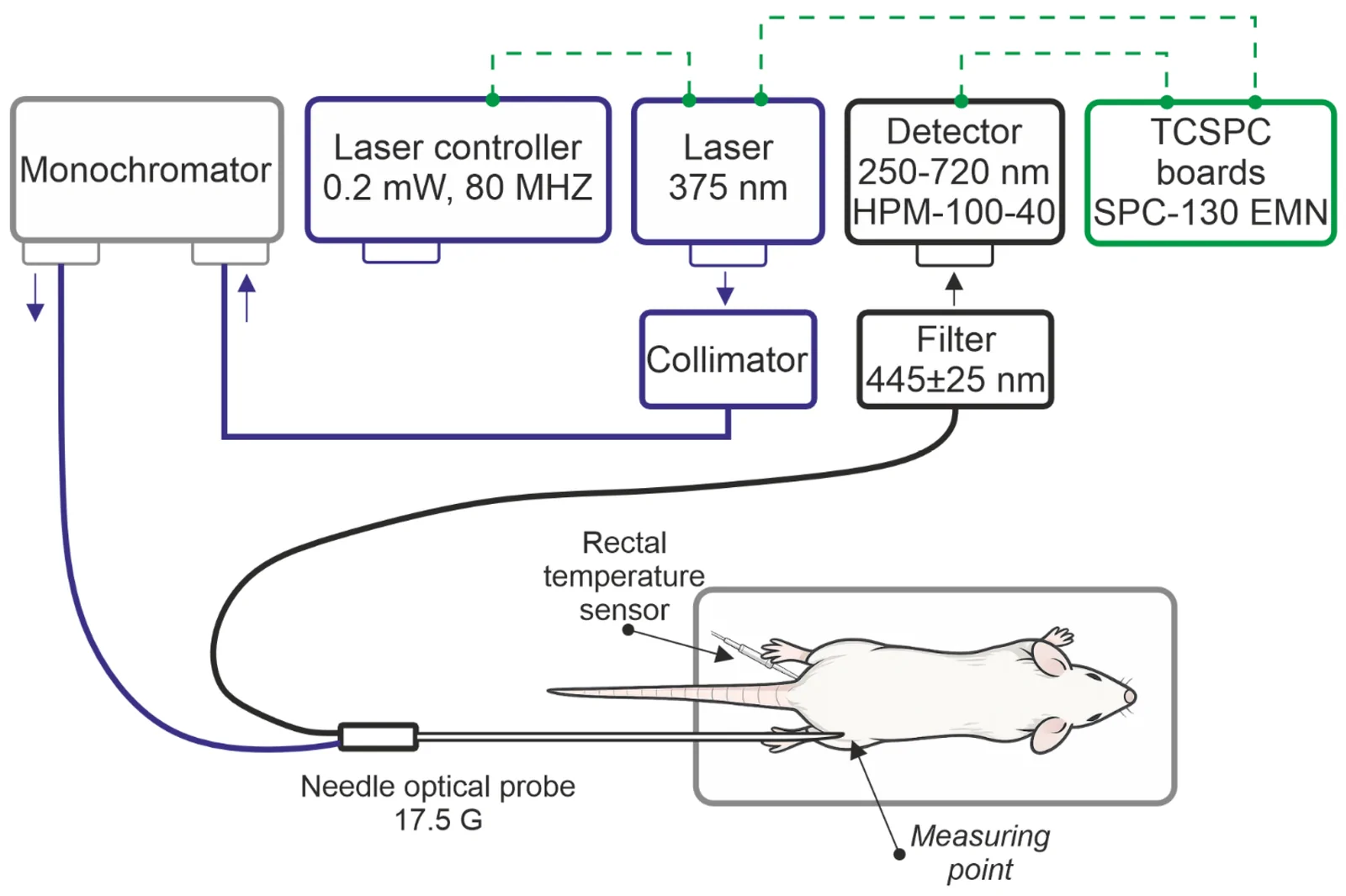
Experimental equipment and design.

To measure the fluorescence lifetime parameters, we used the time-correlated single photon counting (TCSPC) system based on the SPC-130-EMN photon counting board and HPM-100-40 detectors (Becker & Hickl GmbH, Berlin, Germany). The system is designed for recording the fluorescence of the metabolic coenzyme NADH in its free and protein-bound states [54]. The scheme of the experiment, carried out using a fluorescence lifetime spectroscopy device, is shown in Figure 4.

A BDS-SM-375-FBC-101 375 nm laser source (Becker & Hickl GmbH, Berlin, Germany) with an 80 MHz controller was used for fluorescence excitation. A MonoScan2000 monochromator (OceanOptics, Inc., Dunedin, FL, USA) was used to isolate a narrow-band optical range. A MF445-45 bandpass filter (Thorlabs, Inc., Newton, NJ, USA) with a bandwidth of 445 ± 25 nm was used to isolate the spectral region of NADH fluorescence emission.

The optical power of the UV radiation after the optical fiber was 0.2 mW. The exposure time for measuring a single spectrum was 1.9 s. The radiation delivery and collection systems are designed as a fiber-optic probe with a diameter of 1 mm and a rigid distal end [55,56]. The probe contains 10 fibers: in this experiment, the central fiber was connected to the laser source, while the detector was connected to three peripheral fibers around the central one. The remaining fibers were not used. The probe has a 20° bevel at the distal end, which ensures reliable and maximal contact of the probe surface with the studied tissues during prolonged measurements.

Before insertion, the needle was cleaned with a lens-cleaning wipe moistened with methanol. The needle was then inserted into the deep portion of the gastrocnemius muscle at an angle of approximately 45°. Before the measurements, the performance of the time-resolved FS setup was verified. NADH fluorescence decay spectra were recorded during life in the deep portion of the gastrocnemius muscle of the right hind limb. Immediately after euthanasia, the optical probe was withdrawn from the gastrocnemius muscle of the right hind limb and inserted into the deep portion of the gastrocnemius muscle of the left hind limb. The probe was relocated in order to avoid the influence of the antemortem muscle injury caused by probe insertion on the postmortem fluorescence spectra. Throughout the 6 h of the postmortem period, the probe remained in the gastrocnemius muscle of the left hind limb. At each time point, three consecutive spectra were recorded as technical replicates and averaged. The resulting parameters represented a single observation for each animal at each time point and were used for the subsequent statistical analysis. Measurements were repeated every 30 min. After 6 h, spectral recording was discontinued. At 24 h after euthanasia, additional measurements of the NADH fluorescence decay spectra were performed in the left and right hind limbs.

### 4.6. NADH Fluorescent Lifetime Data Analysis

**Figure 5:**
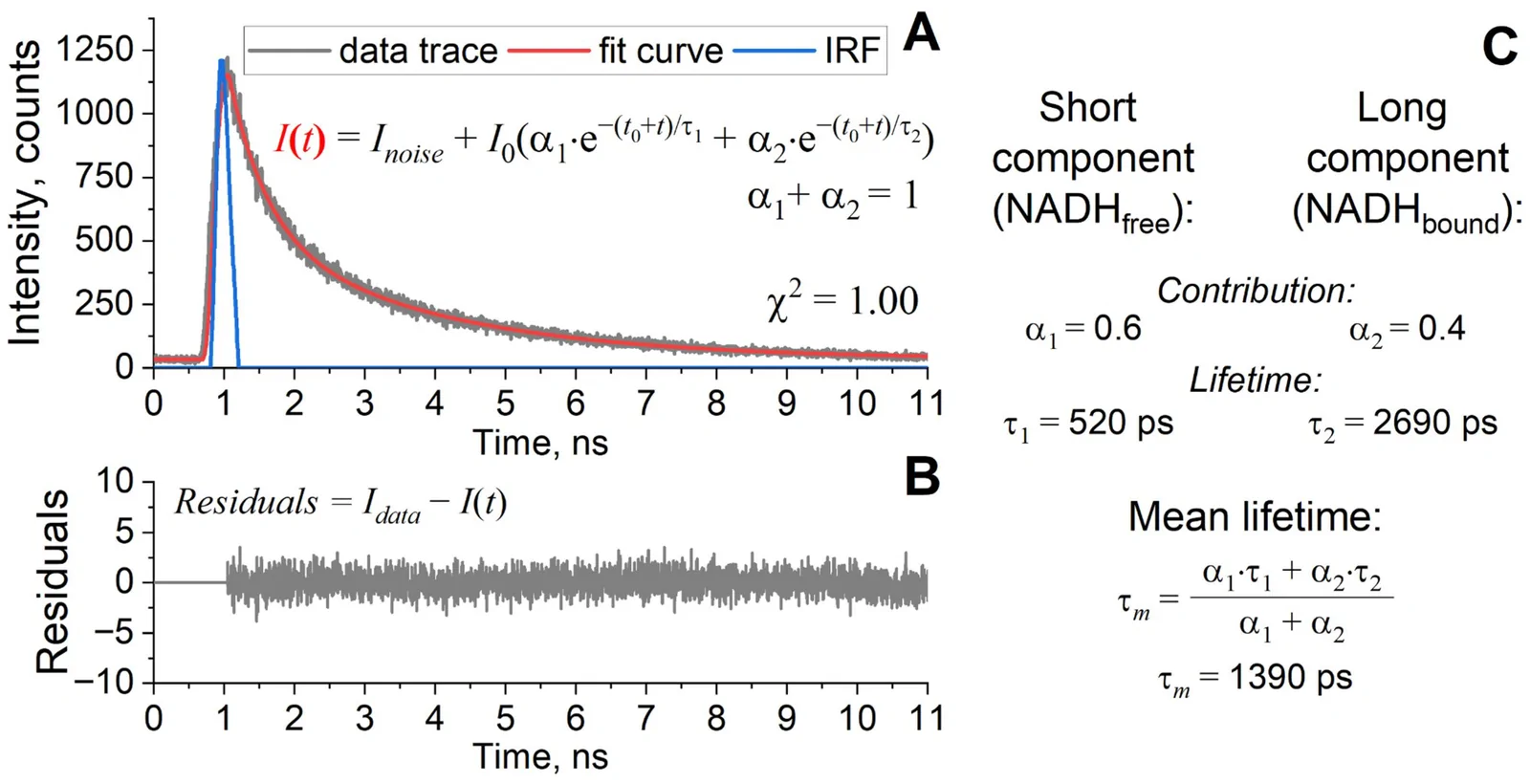
The processing of fluorescence lifetime spectroscopy data: (**A**) measured fluorescence decay data and a biexponential decay model describing the fluorescence decay and instrumental response function (IRF); (**B**) residuals indicating the adequate results of curve fitting in addition to the χ^2^ value; (**C**) fluorescence lifetime components and mean lifetime calculation.

For the quantitative assessment of fluorescence decay, a least-squares curve-fitting algorithm was applied to a two-exponential decay model (Figure 5):

(1) It=Inoise+I0α1e−t0+tτ1+α2e−t0+tτ2,

where *I*(*t*)—fluorescence intensity at time *t* (photon count);

*I*_0_—maximum fluorescence intensity (initial fluorescence intensity after the end of excitation pulse) (photon count);

*I_noise_*—background noise (photon count);

*t_0_*—time delay of maximum fluorescence intensity recording (ns);

*τ*_1_ and *τ*_2_—fluorescence lifetime components (ns):

*τ*_1_—short fluorescence lifetime;

*τ*_2_—long fluorescence lifetime;

*α*_1_ and *α*_2_—relative contributions of the lifetime components (*α*_1_ + *α*_2_ = 1):

*α*_1_—relative contribution of short lifetime component;

*α*_2_—relative contribution of long lifetime component.

A good fit is characterized by a χ^2^ value close to 1.

The mean fluorescence lifetime, which takes into account the relative contribution of fluorescence from each form of NADH, was calculated as:

(2) $$\tau_{m}=\frac{\alpha_{1}\tau_{1}+\alpha_{2}\tau_{2}}{\alpha_{1}+\alpha_{2}}.$$

### 4.7. Statistical Analysis 

Continuous variables were summarized as medians with interquartile ranges (IQRs) and minimum-maximum values. The predictive value of individual predictors was assessed using univariate linear regression models, with PMI as the dependent variable. Model performance was evaluated using the coefficient of determination (R^2^), mean absolute error (MAE), root mean square error (RMSE), and the proportion of observations with an absolute prediction error not exceeding 1 h. Multivariable prediction was performed using partial least squares (PLS) regression. All PLS models were fitted using two latent components and predictors in their original measurement units. Missing values were not imputed. Uncertainty in R^2^, MAE, and RMSE was quantified using cluster bootstrap resampling, with 5000 bootstrap samples and percentile-based 95% confidence intervals (CIs). Paired fluorescence measurements were compared using the two-sided Wilcoxon signed-rank test. To assess the relationship between optical parameters and temperature, partial correlations were calculated while controlling for time after death. All tests were two-sided, and *p*-values < 0.05 were considered statistically significant. Statistical analyses were performed using IBM SPSS Statistics v. 29.0 and Python v3.12. Time-dependent changes were assessed from within-animal trajectories; no repeated-measures test across all postmortem time points was performed, while within-animal pairing was preserved for the antemortem versus 0 h comparison and cluster-bootstrap resampling for predictive-performance confidence intervals was performed at the animal level, with all observations from a selected rat resampled together.

## 5. Conclusions

This exploratory study provides the first description of changes in the NADH fluorescence decay parameters in rat skeletal muscle during the early postmortem period. The parameters τ_1_ and τ_m_, as well as the relative contributions of the biexponential decay components α_1_ and α_2_, showed time-dependent changes during the postmortem period. Among the individual parameters, τ_1_ demonstrated the best predictive performance for estimating the time since death. For PMI prediction within the first 6 h after death, a partial least squares regression model combining α_1_, τ_m_, and the difference between rectal and room temperatures was constructed. The model demonstrated promising predictive performance, with an RMSE of 0.89 h (95% CI: 0.63–0.99), an MAE of 0.68 h (95% CI: 0.49–0.82) and an R^2^ of 0.762 (95% CI: 0.689–0.886); the prediction error did not exceed 1 h in 73.2% of cases. These findings extend our current understanding of the early postmortem changes in skeletal muscle energy metabolism and indicate the potential applicability of NADH fluorescence decay parameters, in combination with temperature variables, for PMI estimation. The data obtained are preliminary and require confirmation in studies with larger samples and under different temperature conditions, followed by validation on human cadaveric material.

## Figures and Tables

**Table 1:** Comparison of NADH fluorescence parameters before death and at 0 h postmortem.

Parameter	Alive	0 h	*p*
τ_1_ (ps)	528.8 (522.4; 540.5)	519.6 (518.1; 522.1)	**0.020**
τ_2_ (ps)	2654.1 (2630.6; 2685.5)	2692.9 (2659.1; 2702.1)	0.064
τ_m_ (ps)	1346.1 (1285.2; 1402.9)	1355.0 (1271.0; 1380.5)	0.375
α_2_ (%)	38.31 (36.31; 39.80)	38.24 (34.60; 39.21)	0.193
α_1_ (%)	61.69 (60.20; 63.69)	61.76 (60.79; 65.40)	0.193
Fluorescence intensity (photons)	684,214 (569,762; 1,050,367)	697,797 (630,376; 1,137,221)	1.000

Data are presented as Me (Q1; Q3). The statistically significant results are highlighted in bold.

**Table 2:** Partial correlations between NADH fluorescence parameters and temperature variables adjusted for PMI.

NADH Fluorescence Parameter	Rectal t. r	*p*	Hind-Limb Surface t. r	*p*	Room t. r	*p*
τ_1_ (ps)	**−0.260**	**0.003**	**−0.407**	**<0.001**	**−0.392**	**<0.001**
τ_2_ (ps)	0.002	0.986	0.019	0.835	−0.091	0.309
τ_m_ (ps)	−0.114	0.206	0.042	0.652	−0.096	0.285
α_2_ (%)	−0.103	0.253	0.060	0.522	−0.072	0.425
α_1_ (%)	0.103	0.253	−0.060	0.522	0.072	0.425
Fluorescence intensity (photons)	**−0.298**	**<0.001**	**−0.265**	**0.004**	**−0.406**	**<0.001**

The statistically significant results are highlighted in bold.

**Table 3:** Performance of individual predictors in univariate analysis and PLS models for estimating the postmortem interval (0–24 h).

Predictor	*n*	R^2^ (95% CI)	MAE, h (95% CI)	MAE, min (95% CI)	RMSE, h (95% CI)	Absolute Error ≤ 1 h, *n* (%)
τ_1_ (ps)	140	0.267 (0.111–0.478)	2.80 (2.20–3.18)	167.8 (131.9–190.8)	4.88 (4.12–5.38)	
τ_m_ (ps)	140	0.207 (0.132–0.376)	3.38 (3.08–3.45)	202.7 (184.7–207.0)	5.08 (4.50–5.31)	
α_2_ (%)	140	0.197 (0.124–0.356)	3.36 (3.05–3.44)	201.4 (182.9–206.6)	5.11 (4.58–5.34)	
α_1_ (%)	140	0.197 (0.126–0.361)	3.36 (3.02–3.44)	201.4 (181.3–206.4)	5.11 (4.56–5.33)	
Fluorescence intensity (photons)	140	0.012 (0.000–0.067)	3.24 (3.20–3.37)	194.3 (191.8–201.9)	5.67 (5.51–5.70)	
τ_2_ (ps)	140	0.008 (0.004–0.171)	3.22 (3.12–3.30)	193.2 (187.3–198.2)	5.68 (5.19–5.69)	
PLS optical model (α_1_; τ_m_)	140	0.209 (0.149–0.394)	3.38 (3.03–3.46)	202.6 (181.6–207.4)	5.07 (4.44–5.26)	26/140 (18.6%)

MAE, mean absolute error; RMSE, root mean square error. The fluorescence parameters included in the PLS models were not significantly correlated with the temperature variables.

**Table 4:** Performance of individual predictors in univariate analysis and PLS models for estimating the postmortem interval (0–6 h).

Predictor	*n*	R^2^ (95% CI)	MAE, h (95% CI)	MAE, min (95% CI)	RMSE, h (95% CI)	Absolute Error ≤ 1 h, *n* (%)
τ_1_ (ps)	130	0.319 (0.173–0.509)	1.28 (1.05–1.46)	76.6 (63.2–87.7)	1.54 (1.31–1.70)	
τ_m_ (ps)	130	0.117 (0.059–0.249)	1.48 (1.32–1.56)	89.0 (79.1–93.3)	1.76 (1.62–1.81)	
α_2_ (%)	130	0.114 (0.053–0.250)	1.48 (1.31–1.55)	88.8 (78.7–93.3)	1.76 (1.62–1.82)	
α_1_ (%)	130	0.114 (0.053–0.250)	1.48 (1.31–1.55)	88.8 (78.7–93.3)	1.76 (1.62–1.82)	
Fluorescence intensity (photons)	130	0.002 (0.000–0.032)	1.62 (1.59–1.62)	97.0 (95.2–97.2)	1.87 (1.84–1.87)	
τ_2_ (ps)	130	0.006 (0.002–0.123)	1.62 (1.51–1.62)	97.0 (90.2–97.1)	1.87 (1.75–1.87)	
PLS optical model (α_1_; τ_m_)	130	0.117 (0.069–0.282)	1.48 (1.27–1.55)	89.1 (76.3–93.0)	1.76 (1.59–1.81)	47/130 (36.2%)
PLS combined model (α_1_; τ_m_; ΔT)	123 *	0.762 (0.689–0.886)	0.68 (0.49–0.82)	40.9 (29.6–49.3)	0.89 (0.63–0.99)	90/123 (73.2%)

MAE, mean absolute error; RMSE, root mean square error; PLS, partial least squares; ΔT, difference between rectal and room temperatures. The PLS optical model included α_1_ and τ_m_, whereas the combined PLS model additionally included ΔT. The PMI estimation equation was as follows: PMI (h) = 7.1210 − 0.02379 × α_1_ (%) + 0.000891 × τ_m_ (ps) − 0.67522 × ΔT, where ΔT was calculated as T_rectal_ (°C)–T_room_ (°C). * The reduction from 130 to 123 observations resulted from seven observations with incomplete temperature data required to calculate ΔT: ambient temperature was unavailable for Rat 1 at 0, 0.5, and 1 h; rectal temperature was unavailable for Rat 2 at 5, 5.5, and 6 h; and both temperatures were unavailable for Rat 4 at 0.5 h. No imputation was performed.

## Data Availability

The original contributions presented in this study are included in the article/Supplementary Materials. Further inquiries can be directed to the corresponding authors.

## Supplementary Materials

The following supporting information can be downloaded at: https://www.mdpi.com/article/10.3390/ijms27188094/s1.

## Appendix A

### Appendix A.1

**Table A1:** Descriptive statistics of NADH fluorescence decay parameters, fluorescence intensity, and temperature measurements in rat skeletal muscle before death and at each postmortem time point.

Parameter	Alive	0 h	0.5 h	1 h	1.5 h	2 h	2.5 h	3 h
**τ_1_ (ps)**	*n =* 10 528.8 (521.4; 541.1) 515.3–584.3	*n =* 10 519.6 (517.1; 523.8) 504.8–528.5	*n =* 10 518.1 (512.6; 522.8) 504.7–531.7	*n =* 10 519.5 (515.9; 527.1) 513.3–530.3	*n =* 10 523.1 (521.9; 527.1) 519.5–530.1	*n =* 10 525.5 (519.2; 532.4) 517.4–538.1	*n =* 10 527.4 (525.2; 533.2) 517.0–535.6	*n =* 10 528.1 (524.7; 532.2) 519.3–541.0
**τ_2_ (ps)**	*n =* 10 2654.1 (2630.6; 2685.5) 2443.1–2708.0	*n =* 10 2692.9 (2659.1; 2702.1) 2618.9–2721.5	*n =* 10 2687.2 (2655.8; 2708.4) 2594.0–2734.5	*n =* 10 2699.4 (2650.9; 2719.0) 2584.2–2748.5	*n =* 10 2701.2 (2658.6; 2719.0) 2564.6–2770.6	*n =* 10 2704.1 (2663.8; 2716.8) 2612.1–2734.7	*n =* 10 2703.8 (2668.8; 2712.7) 527.7–2746.7	*n =* 10 2690.7 (2669.9; 2719.0) 2647.2–2726.0
**τ_m_ (ps)**	*n =* 10 1346.1 (1280.1; 1415.6) 1228.9–1440.6	*n =* 10 1355.0 (1215.6; 1382.9) 1104.9–1409.5	*n =* 10 1331.1 (1202.8; 1376.4) 1117.8–1392.8	*n =* 10 1334.7 (1197.2; 1393.0) 1108.9–1404.6	*n =* 10 1326.3 (1212.0; 1390.1) 1119.4–1414.3	*n =* 10 1342.6 (1234.0; 1391.8) 1176.8–1414.9	*n =* 10 1343.8 (1250.2; 1398.4) 1210.2–1414.6	*n =* 10 1354.1 (1272.8; 1395.6) 1222.8–1421.6
**α_2_ (%)**	*n =* 10 38.31 (35.77; 40.34) 34.39–42.84	*n =* 10 38.24 (32.73; 39.30) 28.39–40.67	*n =* 10 36.97 (32.27; 39.18) 28.70–39.48	*n =* 10 36.85 (32.14; 39.45) 28.76–40.20	*n =* 10 36.81 (32.32; 39.36) 29.33–39.66	*n =* 10 37.29 (33.58; 39.43) 31.46–40.34	*n =* 10 37.65 (34.02; 39.54) 32.40–40.52	*n =* 10 38.03 (35.03; 39.49) 32.35–40.79
**α_1_ (%)**	*n =* 10 61.69 (59.66; 64.23) 57.16–65.61	*n =* 10 61.76 (60.70; 67.27) 59.33–71.61	*n =* 10 63.03 (60.82; 67.73) 60.52–71.30	*n =* 10 63.15 (60.55; 67.86) 59.80–71.24	*n =* 10 63.19 (60.64; 67.68) 60.34–70.67	*n =* 10 62.71 (60.57; 66.42) 59.66–68.54	*n =* 10 62.35 (60.46; 65.98) 59.48–67.60	*n =* 10 61.97 (60.51; 64.97) 59.21–67.65
**Fluorescence intensity** **(photons)**	*n =* 10 684,214 (538,600; 1,133,062) 447,637–1,437,033	*n =* 10 697,797 (610,449; 1,278,868) 217,938–1,384,168	*n =* 10 811,327 (670,108; 1,195,587) 251,144–1,544,618	*n =* 10 834,239 (677,456; 1,220,121) 274,184–1,396,525	*n =* 10 861,554 (651,324; 1,189,485) 325,503–1,385,091	*n =* 10 859,688 (677,696; 1,179,465) 364,908–1,444,683	*n =* 10 884,787 (673,780; 1,189,309) 376,071–1,455,171	*n =* 10 932,561 (753,753; 1,265,440) 359,475–1,492,529
**T rectal, °C**	*n =* 0 Not available	*n =* 10 35.3 (34.0; 35.6) 33.5–37.0	*n =* 9 33.9 (32.3; 34.4) 31.3–36.1	*n =* 10 32.8 (31.8; 33.1) 30.4–33.8	*n =* 10 31.6 (31.2; 32.1) 29.8–32.2	*n =* 10 30.8 (30.6; 31.0) 29.1–31.1	*n =* 10 30.0 (29.8; 30.2) 28.5–30.6	*n =* 10 29.4 (29.1; 29.7) 28.0–29.8
**T paw, °C**	*n =* 0 Not available	*n =* 8 31.1 (29.8; 31.2) 28.3–31.6	*n =* 7 28.1 (27.3; 28.7) 25.7–29.0	*n =* 8 27.6 (26.7; 27.9) 26.1–28.3	*n =* 8 27.1 (26.1; 27.6) 25.0–28.1	*n =* 10 26.9 (26.0; 27.5) 24.2–28.6	*n =* 10 26.4 (25.6; 27.1) 24.0–28.2	*n =* 10 26.0 (25.4; 26.8) 23.6–27.8
**T room, °C**	*n =* 0 Not available	*n =* 9 24.6 (24.4; 25.6) 24.0–26.0	*n =* 8 24.6 (24.1; 25.6) 22.5–25.9	*n =* 9 24.1 (24.0; 25.9) 23.9–26.0	*n =* 10 24.3 (24.1; 25.9) 23.3–26.1	*n =* 10 24.1 (23.9; 25.8) 23.1–26.1	*n =* 10 23.9 (23.4; 25.4) 22.9–26.1	*n =* 10 23.9 (23.4; 25.1) 22.8–26.1
**Parameter**		**3.5 h**	**4 h**	**4.5 h**	**5 h**	**5.5 h**	**6 h**	**24 h**
**τ_1_ (ps)**		*n =* 10 525.0 (521.9; 531.1) 508.0–535.4	*n =* 10 527.4 (521.9; 534.3) 515.8–537.2	*n =* 10 530.0 (524.5; 536.5) 517.9–538.9	*n =* 10 531.1 (526.1; 536.4) 524.6–541.6	*n =* 10 533.2 (528.6; 535.7) 518.8–541.4	*n =* 10 532.2 (529.8; 534.5) 524.2–545.7	*n =* 10 536.3 (529.8; 544.4) 526.0–604.7
**τ_2_ (ps)**		*n =* 10 2686.7 (2664.5; 2704.7) 2640.8–2738.4	*n =* 10 2697.4 (2673.3; 2708.6) 2628.6–2755.8	*n =* 10 2698.9 (2660.8; 2723.7) 2623.2–2728.6	*n =* 10 2708.0 (2696.4; 2718.0) 2641.1–2763.7	*n =* 10 2709.8 (2700.4; 2720.3) 2675.1–2762.4	*n =* 10 2717.6 (2700.9; 2730.8) 2668.0–2775.3	*n =* 10 2727.7 (2717.7; 2737.5) 2685.2–2767.2
**τ_m_ (ps)**		*n =* 10 1354.1 (1274.2; 1400.1) 1219.1–1408.8	*n =* 10 1346.8 (1278.1; 1400.3) 1214.3–1415.2	*n =* 10 1378.2 (1275.4; 1421.8) 1232.4–1444.1	*n =* 10 1391.3 (1327.1; 1423.6) 1269.0–1464.0	*n =* 10 1377.9 (1321.8; 1421.9) 1299.6–1447.2	*n =* 10 1400.7 (1337.5; 1429.9) 1285.2–1458.8	*n =* 10 1472.4 (1423.8; 1486.3) 1417.3–1513.1
**α_2_ (%)**		*n =* 10 38.01 (35.41; 39.92) 32.44–40.73	*n =* 10 37.99 (35.12; 39.80) 32.27–41.40	*n =* 10 38.82 (35.29; 40.49) 33.79–41.43	*n =* 10 39.76 (36.59; 40.79) 34.99–41.51	*n =* 10 39.18 (36.21; 40.67) 35.69–41.72	*n =* 10 39.64 (36.64; 40.82) 34.80–41.85	*n =* 10 42.23 (40.79; 43.22) 39.12–44.41
**α_1_ (%)**		*n =* 10 61.99 (60.08; 64.59) 59.27–67.56	*n =* 10 62.01 (60.20; 64.88) 58.60–67.73	*n =* 10 61.18 (59.51; 64.71) 58.57–66.21	*n =* 10 60.23 (59.21; 63.41) 58.49–65.01	*n =* 10 60.82 (59.33; 63.79) 58.28–64.31	*n =* 10 60.36 (59.18; 63.36) 58.15–65.20	*n =* 10 57.77 (56.78; 59.21) 55.59–60.88
**Fluorescence intensity** **(photons)**		*n =* 10 931,063 (730,565; 1,263,394) 353,757–1,451,109	*n =* 10 920,809 (692,586; 1,255,122) 355,817–1,470,860	*n =* 10 955,739 (722,377; 1,176,300) 355,352–1,484,714	*n =* 10 936,790 (609,198; 1,183,153) 388,163–1,479,604	*n =* 10 944,301 (542,666; 1,192,103) 393,965–1,470,365	*n =* 10 907,988 (610,868; 1,194,392) 336,416–1,477,245	*n =* 10 742,105 (632,527; 855,451) 483,796–1,023,675
**T rectal, °C**		*n =* 10 29.0 (28.3; 29.2) 27.5–29.6	*n =* 10 28.5 (28.0; 28.8) 26.9–29.3	*n =* 10 28.0 (27.5; 28.5) 26.2–29.1	*n =* 9 27.5 (26.9; 27.9) 26.1–29.0	*n =* 9 27.3 (26.7; 27.6) 25.8–28.8	*n =* 9 27.1 (26.4; 27.4) 25.9–28.8	*n =* 0 Not available
**T hind limb, °C**		*n =* 10 25.8 (25.1; 26.6) 23.0–27.7	*n =* 10 25.6 (25.2; 26.4) 22.8–27.6	*n =* 10 25.4 (24.6; 26.1) 23.1–27.2	*n =* 10 25.2 (24.4; 26.1) 23.1–27.3	*n =* 9 24.9 (24.6; 25.6) 23.5–26.6	*n =* 9 24.9 (24.2; 25.4) 23.1–26.3	*n =* 0 Not available
**T room, °C**		*n =* 10 23.6 (23.3; 25.1) 22.6–26.0	*n =* 10 23.9 (23.2; 25.1) 22.6–26.2	*n =* 10 23.6 (23.2; 24.9) 22.8–26.1	*n =* 10 23.6 (23.3; 24.6) 22.9–26.3	*n =* 10 23.9 (23.3; 24.5) 23.3–26.1	*n =* 10 23.6 (23.3; 24.2) 21.7–26.2	*n =* 0 Not available

**Note:** Each cell presents N, median, (Q1; Q3), and minimum–maximum. τ_1_, short-lifetime component; τ_2_, long-lifetime component; τ_m_, mean fluorescence lifetime; α_1_ and α_2_, relative contributions of the short- and long-lifetime components, respectively; T rectal, rectal temperature; T paw, paw temperature; T room, room temperature.

### Appendix A.2

**Table A2:** Comparison of NADH fluorescence parameters between the right and left hind limbs at 24 h postmortem.

Parameter	Right Hind Limb	Left Hind Limb	*p*
τ_1_ (ps)	540.5 (534.7; 544.1)	528.1 (526.0; 539.3)	**0.049**
τ_2_ (ps)	2736.4 (2724.7; 2752.2)	2726.5 (2702.6; 2736.0)	0.432
τ_m_ (ps)	1469.6 (1445.6; 1489.7)	1469.9 (1456.5; 1495.1)	1.000
α_2_ (%)	42.31 (41.46; 42.98)	42.81 (42.38; 43.13)	0.322
α_1_ (%)	57.69 (57.02; 58.54)	57.19 (56.87; 57.62)	0.322
Fluorescence intensity (photons)	594,353 (546,984; 789,864)	699,240 (538,539; 1,030,794)	0.492

Data are presented as Me (Q1; Q3). Between-limb comparisons were performed using the two-sided Wilcoxon signed-rank test. Statistically significant results are highlighted in bold.
